# Operating Theatre Ventilation Systems and Their Performance in Contamination Control: “At Rest” and “In Operation” Particle and Microbial Measurements Made in an Italian Large and Multi-Year Inspection Campaign

**DOI:** 10.3390/ijerph17197275

**Published:** 2020-10-05

**Authors:** Francesco Romano, Samanta Milani, Roberto Ricci, Cesare Maria Joppolo

**Affiliations:** 1 Dipartimento di Energia, Politecnico di Milano, 20156 Milan, Italy; samanta.milani@polimi.it (S.M.); cesare.joppolo@polimi.it (C.M.J.); 2 Biologist–OT Validation Specialist, 60035 Jesi, Italy; r.ricci70@gmail.com

**Keywords:** operating theatres, ventilation systems, particle contamination, microbiological contamination, unidirectional airflow, mixing airflow

## Abstract

In Operating Theatres (OT), the ventilation system plays an important role in controlling airborne contamination and reducing the risks of Surgical Site Infections (SSIs). The air cleanliness is really crucial in this field and different measurements are used in order to characterize the situation in terms of both airborne microbiological pollutants and particle size and concentration. Although the ventilation systems and airborne contamination are strictly linked, different air diffusion schemes (in particular, the Partial Unidirectional Airflow, P-UDAF, and the Mixing Airflow, MAF) and various design parameters are used, and there is still no consensus on real performance and optimum solutions. This study presents measurements procedures and results obtained during Inspection and Periodic Performance Testing (1228 observations) in a large sample of Italian OTs (175 OTs in 31 Italian hospitals) in their operative life (period from 2010 to 2018). The inspections were made after a cleaning procedure, both in “at-rest” conditions and “in operation” state. Inert and microbial contamination data (in air and on surfaces) are analyzed and commented according to four relevant air diffusion schemes and design classes. Related data on Recovery Time (RT) and personnel presence were picked up and are commented. The results confirm that the ventilation systems are able to maintain the targeted performance levels in the OT operative life. However, they attest that significant differences in real OT contamination control capabilities do exist and could be ascribed to various design choices and to different operation and maintenance practices. The study shows and confirms that the air diffusion scheme and the design airflow rate are critical factors. Beside large variations in measurements, the performance values, in terms of control of airborne particle and microbial contamination (in air and on surfaces), for P-UDAF systems are better than those that were assessed for the MAF air diffusion solution. The average performances do increase with increasing airflows, and the results offer a better insight on this relationship leading to some possible optimization.

## 1. Introduction

Surgery operations are one of the most vulnerable procedures for infections in operating theatres. It is known that patients with low immune protection are prone to infections. Air is considered to be a vector of pathogens and an important cause of surgical site infections (SSI) [1]. The latter refer to infections that are located on the patient’s wound after surgical activity. They are associated with increased morbidity, longer hospital stays, and increased costs for communities [2,3,4,5,6,7]. Contamination in the surgical field occurs directly from human contamination and indirectly through instruments, gloves, and components. However, both direct and indirect contamination depend on air quality and ventilation system, as well as on medical team behaviors, patients’ health, surgical procedures, etc. [8,9,10]. A good level of air cleanliness inside the operating theatre (OT) can prevent the number of SSIs by controlling the level of total airborne contamination (inert and microbiological) through proper air ventilation. In 2017 in 12 EU Member States and 1 EEA country, the percentage of SSIs varied from 0.5% to 10.1%, depending on the type of surgical procedure, based on a work done by the ECDC for nine different types of surgical procedures [11].

The relationship between wound infection and airborne contamination has been studied since the 1940s [12,13,14,15,16], with increasing interest on the analysis of operating theatres with different types of ventilation systems, on the effect on cross-contamination, and on viable airborne reduction [17]. Based on those studies, two main types of ventilation for OT environments have been identified: mixing ventilation airflow (MAF) and partial unidirectional airflow (P-UDAF). The mixing system dilutes airborne contamination by turbulent mixing between supplied clean sterile air and contaminated room air. By contrast, the P-UDAF system, due to its low turbulence flow generally directed downwards, moves contaminants away from the OT protected area (around the surgical table) and reduces the risk of cross-contamination from the outer zone where airflow conditions are not unidirectional. The MAF system with terminal mixing diffusers and the P-UDAF system with unidirectional ceiling systems supply sterile air cleaned by high efficiency HEPA air filters. Types of ventilation systems other than those described are not commonly used.

Establishing which of the two systems was the best has been a controversial and contradictory issue for many years [18]. In several studies where viable contamination was measured, P-UDAF appears to have lower contamination values than the MAF system [19,20,21,22,23,24,25,26]. However, some clinical registry studies conducted in recent years suggested that the risk of SSIs after surgery in P-UDAF systems is equal or even higher than in MAF [1,27,28,29,30]. The World Health Organization (WHO) suggests the use of MAF systems for specific type of surgery [9]. According to many studies, conclusions based on ventilation data from national registries and epidemiological studies should not be interpreted without considering data inaccuracy [16]. In this regard, the study that was carried out by Popp et al. [31] confirmed that inaccuracies and a lack of information in compiling the SSI national registry may significantly diverge the final results from reality. The conclusions of this paper show that P-UDAF systems perform better in terms of airborne contamination and SSIs protection in OT environments.

The efficiency of a ventilation system is influenced by multiple factors, such as the position of the operating table, the operating team, the surgical lamps, the type of personnel clothing systems, the surgical equipment [18,32,33,34,35], and the frequency of door opening. The surgical procedures [8] and the local risk assessment adopted for preventing SSI [18] also present particular relevance, as confirmed by recent studies on MAF [1,12,36,37] and P-UDAF systems [1,8,12,38,39,40,41,42]. A comprehensive overview of the SSI prevention guidelines has been carried out [43] in order to give holistic tentative indications on risks and factors (e.g., patient, medical team, surgeries, procedures, HVAC systems, etc.) that may cause SSIs through contamination.

In addition to the numerous studies conducted on ventilation efficiency, OT performance is often evaluated in terms of microbiological and inert contamination of air and surfaces [44,45] according to the guidelines and to the limit values used in cleanroom environments for pharmaceutical production. These environments conform to Good Manufacturing Practice Guidelines (GMPs) and ISO 14644:1 standards or national guidelines [46,47,48] and they are classified “at-rest” and “in operation” occupancy states. A similar approach is also used by the Italian guideline for environmental monitoring of operating theaters, as issued by INAIL [49].

These guidelines and standards specify the air cleanliness level in terms of total and biological airborne particles. The total particles are generally classified in the range from 0.5 to 20 microns, bio particles (known as bacteria-carrying particles) are mostly in the range from five to 60 microns, while bacteria are in the range from one to 15 microns [9]. The main source of contamination within OTs are human activities, clothing, and equipment.

Previous studies reported here concerned the interaction between type of ventilation system and SSI events occurring after surgical operations in the “operational” state of an OT, with the data and results mainly based on epidemiological studies and national infection databases, and not on actual “on- site” measurements in OTs during surgical activities.

At the moment, there is a lack of studies on OT contamination cleanliness levels in “at-rest” and in “operational” not simulated state: few studies have been conducted in those conditions giving much attention to the Total Viable Count (TVC) [50,51] or Total Bacterial Count (TBC) [26] in the OTs that were equipped with different ventilations systems more than assessing the technical HVAC parameters that may influence the OT contamination. Usually, an operating theater is cleaned before each surgery in order to keep the total and microbial particle concentration levels below a fixed threshold before each operation begins. Compliance with these limits under all occupational states can be considered to be a key parameter in assessing and maintaining the contaminant concentration below the prescribed limits.

An eight-year on-site measurement campaign has been conducted in 175 OTs of 31 Italian hospitals in order to quantify the cleanliness level and the performance of operating theaters depending on the ventilation systems, leading to a database of 1228 complete inspections.

Two type of ventilations systems, P-UDAF and MAF, have been monitored in “at-rest” and “operational” occupational states according to the inspection protocols that were suggested by INAIL (Istituto Nazionale per l’Assicurazione contro gli Infortuni sul Lavoro, “National Institute for Insurance agains Accidents at Work”) guidelines [49] and EU-GMP (European Union’s Good Manufacturing Practice) [52] for total and microbiological contamination in air and on surfaces. In addition, technical parameters, such as airflow rate, recovery time, and number of people, were monitored. A further aim is to analyze which ventilation system can guarantee the compliance with the imposed cleanliness limits. To our best knowledge, this is the first inspection study in hospital, planned and carried out with this objective in Italy, due to the large sample set and long-time span. The knowledge gained by this study on contamination control in OTs can help hospitals and engineers to select and operate OT ventilation systems and OT procedures properly, ensuring low contamination levels, low infection rates, and safer working conditions.

## 2. Materials and Methods

The monitoring campaign of this study encompasses the whole Inspection and Periodic Performance Testing (IPPT) process, includes measurements of airborne particle and microbiological pollutant concentrations, and has been carried out in a large sample of Italian operating theatres. The measurements were periodically repeated over a long period extending from 2010 to 2018. The total number of complete IPPTs is equal to 1228; the inspections were made in 175 OTs that were situated in 31 hospitals throughout the Italian peninsula. The periodic inspections had the role of assessing the status of air cleanliness and of ventilation system, and proving continued compliance to the classification of the Italian INAIL Guidelines OT performance requirements [49]. The IPPT procedure has been based on INAIL GL Guidelines [49], and on pertinent ISO 14644-1 [46], ISO 14698-1 [53], and EU-GMP [52] for particle and microbiological contamination in air and on surfaces. Each inspection included a full set of measurements. The classification of air cleanliness in terms of airborne particles concentration was carried out under “at-rest” conditions. Microbiological air sampling was made under “at-rest” and “in operation” (real surgery) conditions. Recovery time, airflow rates, air velocities, and differential pressures were measured under “at-rest” conditions, and other data (e.g., number of people in OT) were monitored “in operation”. It is worth highlighting that the methodology, the procedures, and the instruments applied to all IPPTs during the entire measurement campaign has been maintained unchanged, thus assuring accurate, consistent, and comparable results. In “at-rest conditions” measurements have been carried out after a complete OT cleaning procedure, which is carried out before and after each real surgery. The evaluation of the OT cleaning procedure was out of the scope of this research work.

The measurements are presented by distinguishing between OTs adopting the P-UDAF or MAF scheme and dividing these two main categories according to relevant differences in the ventilation system required performance and in air diffusion design choices. Therefore, the four categories of OT ventilation are here listed:

- Type A—Partial UniDirectional AirFlow (P-UDAF)—designed (airflow rate and ceiling area) to achieve an ISO 5 air cleanliness class in “at-rest” condition;
- Type B—Partial UniDirectional AirFlow (P-UDAF)—designed (airflow rate and ceiling area) to achieve an ISO 7 air cleanliness class in “at-rest” condition;
- Type C—Mixing Air Flow (MAF)—designed to achieve an ISO 7 air cleanliness class in “at-rest” condition and adopting high-wall supply grilles; and,
- Type D—Mixing Air Flow (MAF)—designed to ISO 7 air cleanliness in “at-rest” condition and adopting ceiling air diffusers.

ISO air cleanliness classes are evaluated according to ISO 14644-1:2001-2015 [46]. Table 1 describes the type of measurements and sampling points chosen for the environmental monitoring campaign.

Recovery Time (RT) and ISO class have been measured and calculated according to ISO 14644-3:2005 [47] and ISO 14644-1:2001-2015 [46], respectively. Microbiological air sampling was conducted using an active air sampler (mod. SAS Super ISO 180, VWR International srl, Milano, Italy) with an airflow rate of 180 L per minute (LPM) ± 9 LPM. Sterile TSA (Triptic Soy Agar; VWR International Srl, Milan, Italy) petri dishes with a diameter of 55 mm were used. Under “at-rest” conditions, a total air volume of 1.05 m^3^ was sampled in three separate and sequential series of 350 L each, while, under operating conditions, 200 L were sampled each time, with a hold time before measuring of 5 min. between each consecutive sample, for a minimum of seven series. A new Petri dish was used at each sample run, and the results were averaged per each measurement. Surface microbiological sampling has been made by means of sterile TSA RODAC (Replicate Organism Detection and Counting) plate, 55 mm in diameter, one plate per each of the chosen locations.

The airflow rate, RT, air velocity, temperature, and OT differential pressure were measured under “at-rest” conditions according to ISO 14644-3:2005 [47]. The frequency of staff attendance was monitored during normal surgical operations. The contamination threshold limits were taken from INAIL Guidelines, as shown in Table 2. Measurements in both of the occupational states have been always carried out maintaining the OTs’ doors and windows closed unless emergency events.

Airborne particle threshold limits for OTs, as indicated in INAIL and ISO 14644-1, are 3520 and 352,000 particles/m^3^ (pp/m^3^) for particle diameter ≥0.5 µm (cumulative value) for ISO 5 and ISO 7 Class, respectively. These values are consistent with the threshold limit values imposed by EU-GMP Annex 1 [52]. Table 3 shows EU-GMP limit values for airborne particle and microbial contamination in cleanrooms.

Airborne particle contamination was measured with an optical particle counter (OPC, Lasair III, mod. 350 L, PMS, Boulder, CO, USA). The airflow rate is 50 LPM ± 5%, the detectable particle size channels used were 0.3, 0.5, 1, 5, and 10 µm, with particle counting efficiency equal to 50% for particles ≤0.3 µm, and 100% for particle ≥0.5 µm The calibration of the instrumentation was performed according to ISO 21501-4:2007-2018 [45]. The sampling time and volumes were chosen according to ISO 14644-1:2001-2015.

## 3. Results and Discussion

The monitoring measurements, 1228 complete IPTTs, have been carried out with the aim of assessing the cleanliness performances of the OTs in relation to ventilation systems and other key parameters. The “at-rest” and “operational” occupancy states have been studied in order to better characterize the influence of the ventilation systems. The main results of the experimental campaign are explained in the following paragraphs. The collected data were analyzed with descriptive statistics, resulting in mean, standard deviation, and range for the same OT over time or for each type of OT ventilation system. Continuous variables were compared while using the student’s t-test with 95% confidence level (assuming unknown but equal variances). Multiple population means were compared by analysis of variance (ANOVA) and *p*-values were reported. A low *p*-value indicates a high likelihood of rejecting the null hypothesis of equal means across the populations. A *p*-value < 0.05 is considered to indicate significance. The statistical analysis was performed while using Matlab^®^ (The MathWorks, Natick, MA, USA).

Among the 175 operating theaters under analysis, 58 (33% of the total) OTs had partial vertical unidirectional airflow ventilation (P-UDAF), of which 27 were Type A and 31 Type B, and 117 (67%) had mixing turbulent airflow ventilation (MAF), of which 16 were Type C and 101 Type D. Table 4 shows the average values of the key parameters for OTs by the type of ventilation system.

The OT sizes ranged from 19 to 53.7 m^2^, with a mean of 38.2 m^2^ and volume of 114.1 m^3^. The mean number of air changes per hour (ACH) was 51.2 for Type A, 19 for Type B, 15.1 for Type C, and 18.4 Type D. In all of the OTs, the ventilation systems were equipped with HEPA (High Efficiency Particulate Filters) with a particle filtration efficiency of ≥99.97% for Most Penetrating Particle Size (MPPS) particles. Over the evaluated period, the key performance parameters have shown stable values with some outliers due to occurrences unrelated to standard cleaning and surgical procedures. These data have not been accounted for in this work. Differential pressure between the evaluated OTs and the ancillary areas was always positive, average value of 10 Pa for both typology of ventilation scheme adopted, guaranteeing a correct and safe OTs’ contamination protection from external dirty air infiltration.

### 3.1. OT Contamination Performances in “At-Rest” Conditions

**Figure 1:**
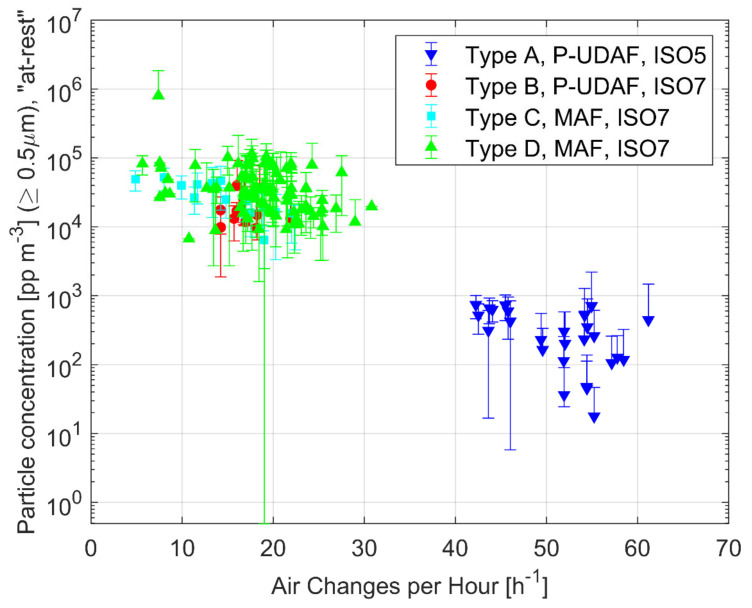
Average airborne particle concentration vs. ACH in “at-rest” conditions for the 175 investigated OTs. Cumulated values for particles ≥0.5 µm.

An influential performance parameter for contamination control in OTs is the level of total airborne particles in “at-rest” condition after the cleaning procedure. This value, besides the quality and the reproducibility of cleaning procedure, could be a useful indication of the ventilation system ability to control airborne contamination below the desired threshold limit. Figure 1 shows the airborne particle concentration values for the OTs under evaluation, being grouped by ventilation type and ISO Class in function of Air Changes per Hour (ACH).

**Figure 2:**
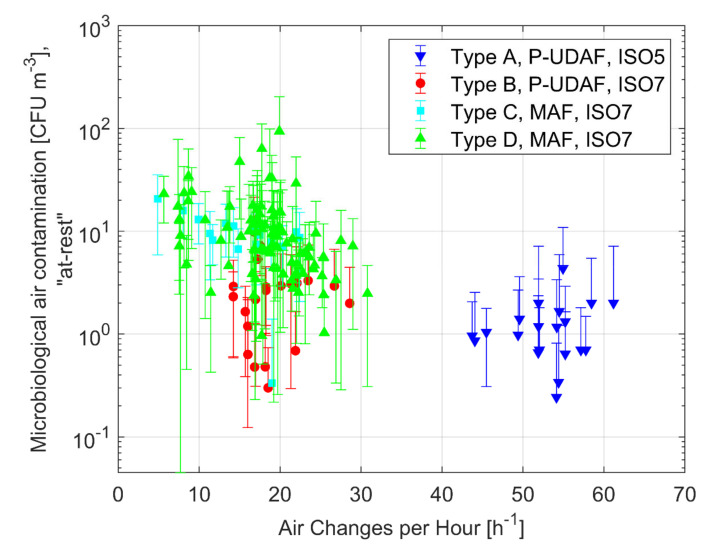
Average microbiological air contamination vs. air changes per hour (ACH) in “at-rest” conditions for the 175 investigated OTs.

The Type A OTs (P-UDAF–ISO 5 design) are offering the best performance in terms of particle concentration control. They process a large air volume uniformly distributed downwards; the concentration of particles ≥0.5 µm in “at-rest” conditions is always far below the limit set for ISO 5 class, with an average values of 326 pp/m^3^, and a highest value that remains below the limit of 3520 pp/m^3^. The P-UDAF Type B OTs, which are designed to obtain an ISO 7 class, are still offering good performance, always below the ISO 7 class limits, and, in comparison to Type A, they significantly decrease the supply airflows. The Type C and D mixing airflow ventilation systems (MAF) show an average concentration below the ISO 7 limit and a quite large performance dispersion. The Type C and D performance in particle concentration control is much lower than the Type A and it could be considered, as can be seen from Table 4 and Figure 1, also worse than the Type B. When comparing Type C with Type D, it appears a better performance of the ceiling air diffusion solution. The *p*-values of the comparison of concentration means are reported in Table 5. It’s observed that MAF and P-UDAF means in ISO class 7 are not significantly different. In particular, P-UDAF Type B and MAF Type C results in *p*-value = 0.997, which implies that, also in this case, the means are not statistically different from each other. In clean and controlled environments, the level of bio-contaminants is related to the total airborne particle concentrations and, in fact, microbes are generally carried by bacteria carrying particles i.e., larger particles that are released by pollutant sources (e.g., skin flakes and respiratory fluid droplets released by humans). Figure 2 shows the level of microbiological airborne contamination under “at-rest” conditions.

The results highlight that the Type A OTs (P-UDAF–ISO 5 design) are also offering the best performance in terms of airborne microbiological concentration control, i.e., an average value of 0.9 CFU/m^3^. Type B OTs, designed to obtain an ISO 7 class, are offering lesser (average concentration 3.3 CFU/m^3^) but quite similar performances. The Type C and D mixing airflow ventilation systems (MAF) show average concentration of 9.9 CFU/m^3^ and 11.8 CFU/m^3^. Once again, it is worth noting both average values and the wide dispersion of results. The comparison of population means presented in Table 5 shows high *p*-values between air distribution patterns of the same type: Type A and Type B have *p*-value = 0.795; Type C and Type D show *p*-value = 0.968. Moreover, although the *p*-value between Type B and Type C is low, it is not possible to reject the null hypothesis. The obtained results are in disagreement with a recent study conducted on the air contamination in orthopedic OTs [51], while well agree with those of similar works conducted in Italian hospitals [8,26] where OTs equipped with unidirectional ventilation schemes always got better air quality than mixing one.

The performance of the ventilation system in controlled environments, such as OT, can also be measured by the Recovery Time (RT). This test, generally carried out in “at-rest” conditions and in non-unidirectional ventilation system, indicates the time interval that is needed for a clean environment to recover the target cleanliness threshold level from a high challenging particle concentration.

**Figure 3:**
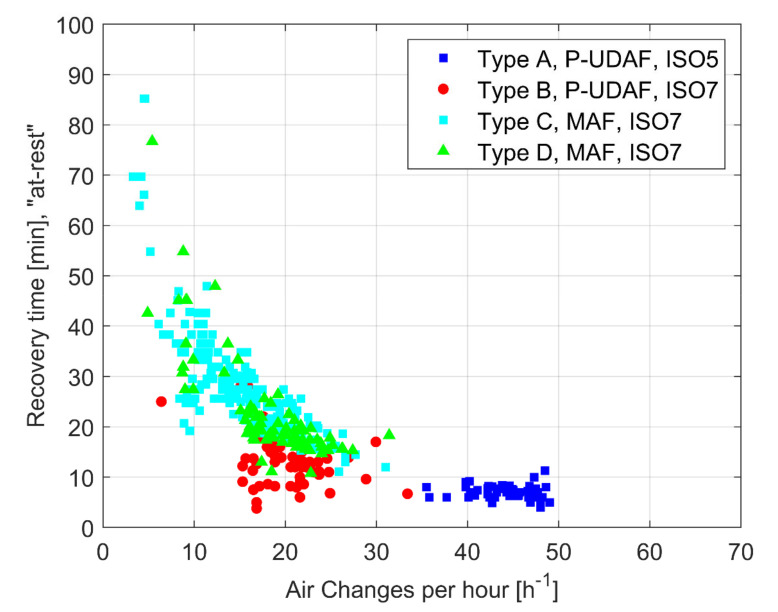
Recovery Time vs. ACH in “at-rest” conditions. Values of the 175 investigated OTs.

The effect of ACH on RT is crucial for P-UDAF (Type A and Type B) systems in ISO 5 and ISO 7 classes (see Figure 3). The influence of ACH on RT, although existent, is little marked for OTs that are equipped with turbulent systems, where the results are widespread. The average RT values span from 7.2 min. to 14 min. for OT ISO 5 Type A and Type B, respectively, and from 28.5 to 23 min. for ISO 7 class Type C and Type D.

### 3.2. OT Contamination Performances in “Operational” Conditions

**Figure 4:**
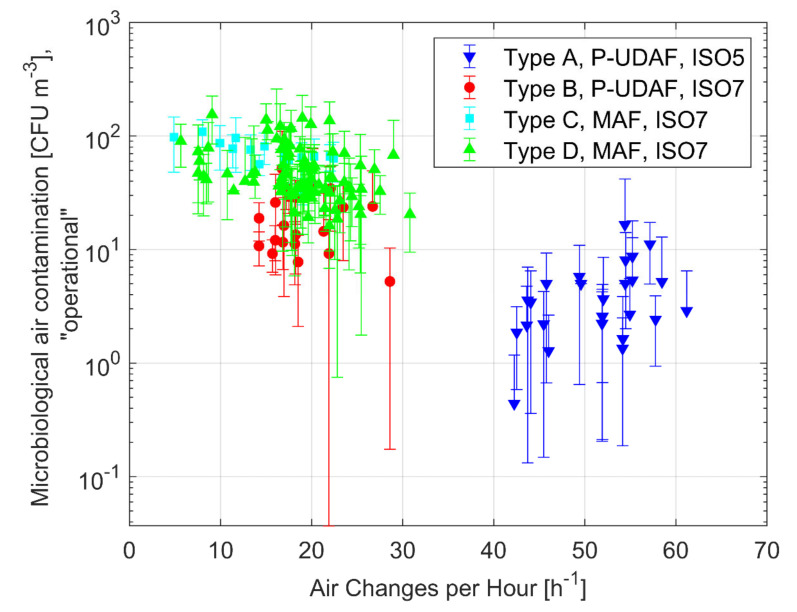
Average microbiological air contamination vs. ACH in “operational” conditions for the 175 investigated OTs.

The influence of the ventilation system in operation conditions is shown in Table 4 and in Figure 4, just with reference to microbiological contaminants. The maximum performance of the P-UDAF system and of its downward piston-like effect are more evident when looking at microbiological contamination level and considering “in operation” instead of “at-rest” conditions. Let us quote from Table 4 that the P-UDAF average values (Type A—5.5 CFU/m^3^; Type B—20.7 CFU/m^3^) are always better than those of MAF systems (Type C—72.8 CFU/m^3^; Type D—55.2 CFU/m^3^).

Let us point out that larger airflows (i.e., larger ACH) could contribute to obtaining a better mixing (ventilation efficiency) in a MAF system and it could maintain optimum velocities in the P-UDAF case, both possibly bringing an indoor air quality improvement. In the studied OTs, as a matter of fact, P-UDAF systems, on average, have 51.2 ACH for ISO Class 5 and 19 ACH for ISO Class 7 classes, and these values are higher than the average ACH of MAF systems, Type C (15.1 ACH) and Type D (18.4 ACH); quite obviously, larger airflows lead towards higher energy consumptions and increased economic costs. It is interesting to look more deeply to the data dispersion (Figure 4); Table 5 presents the comparison of the data samples means in terms of the statistical *p*-values that confirm that the microbiological concentration values between P-UDAF and MAF diffusion schemes are statistically different (*p*-values < 0.05).

In OTs with P-UDAF (Type A—ISO 5; Type B—ISO 7) and, with the highest ACH values, the threshold limits of biological contamination imposed by Italian guidelines in “at-rest” condition (see Table 2) are always met. As the number of ACH decreases and observing the MAF Type C and Type D ventilation systems, the concentration of airborne microbial contaminants in “at-rest” conditions increases and the studied OTs exceed the required limit in 2.6% and 6.1% of the observations, respectively. Under “operational” conditions, the systems respond as in “at-rest” conditions: the lower the ACH, the higher the microbial contamination. EU-GMP Annex 1 for the microbial contamination limits in the “operational” state has strict limits, distinguishing in Grade B (Type A) and Grade C (Type B) air cleanliness level without any distinction among ventilation schemes, on the contrary of INAIL GL, which has larger CFUs concentration limit values for different ventilation schemes. Therefore, according to EU-GMP, it results that the P-UDAF systems meet the threshold limit values 89.2% of the times in ISO 5 Type A class (Grade B) and 98.2% in ISO 7 class Type B (grade C), while MAF diffusion systems meet the EU-GMP threshold limits of 79.7 % and 89.9 % of the times for Type C and Type D (Grade C), respectively. The obtained results agree well with a similar recent work [51], as well as the idea that the actual microbial limit imposed by INAIL GL are too high for mixing airflow OTs.

**Figure 5:**
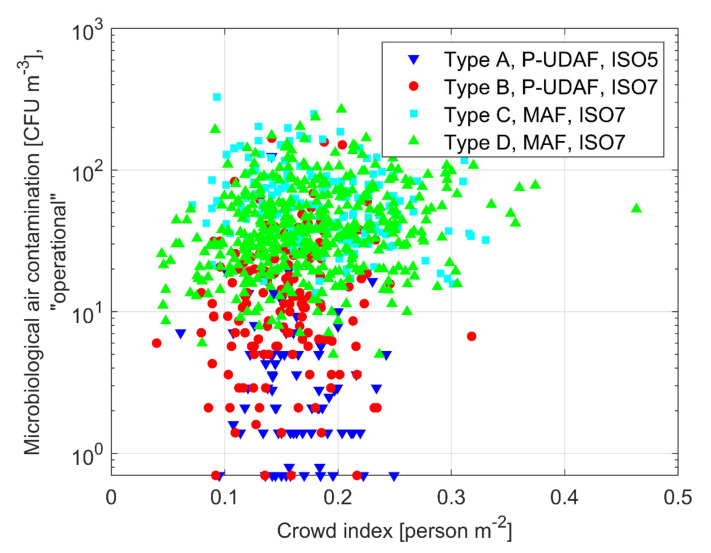
Average microbiological air contamination vs. personnel crowd index in “operational” conditions for different ventilation systems. Values for 175 investigated OTs.

The personnel crown index is another performance parameter that can be related to air contamination in OT environments. Figure 5 shows the microbiological air contamination in “operational” condition as a function of personnel crowd index for OTs equipped with different ventilation systems.

The average concentration of aerial CFUs in relation to the personnel crowd index in “operational” condition varies between 5.5 and 20.7 from ISO 5 class to ISO 7 class in Type A and Type B OTs. In OTs with P-UDAF systems, microbiological contamination varies less than in MAF systems, which is also due to a high ACH and a low variation of the personnel crowd index. In contrast, the low ACHs and the wide variation in the crowd index for MAF OTs (from 0.1 to 0.3) contribute to an increase in CFUs values during surgery agrees with similar multi-year inspection campaigns that were conducted in Italy [8,26], while disagreeing with another similar work in which there was no correlation between crowd index and microbial contamination [51]. Despite the great variability of the crowd index and CFU values, the results shown may also be influenced by other factors, such as type of surgery, technical clothing systems, and patients’ health conditions [26,43,50]

### 3.3. Ancillary Data-Surface Contamination in “At-Rest” Conditions

**Figure 6:**
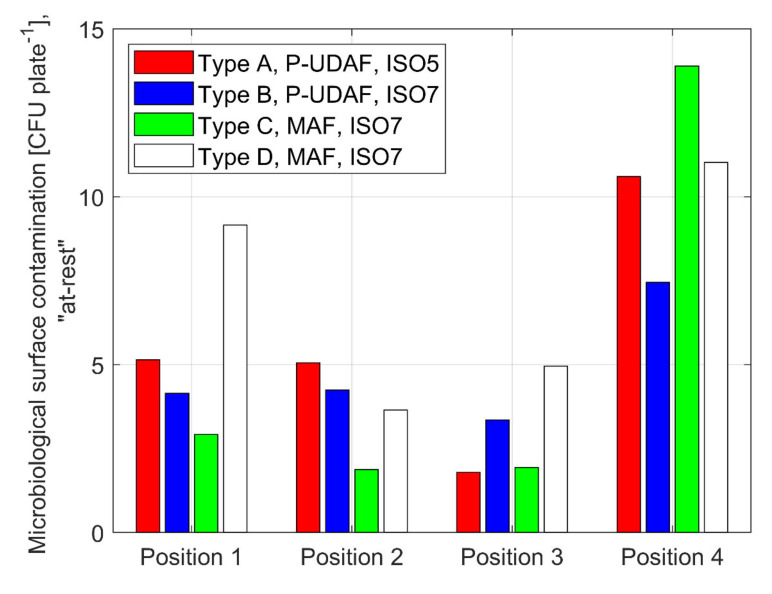
Average microbiological surface contamination in different OT locations under “at rest” conditions after cleaning procedures. Position 1 = surgical table, Position 2 = scialytic lamp, Position 3 = surgical instruments table, Position 4 = anesthesia tower.

The monitoring of surface microbial contamination in “at-rest” conditions is important for interpreting OT cleanliness performance. However, the expected results are more related to the quality of the cleaning process performed before measurements than to the ventilation system itself. The final results of a cleaning procedure are susceptible to human operators, the type of cleaning agents, adopted procedures, and superficial quality of the OTs components and equipment. The high number of variables on which the final cleaning results may depend, in addition to the differences between the procedures in the different hospitals and OTs under study, allows for the authors to consider the surface microbial results as ancillary data, which do not influence the final conclusions. Figure 6 shows the results of all the surface measurements (with sterile TSA RODAC plates) taken on the surgical table and around the critical area.

The results do not show significant differences in averages and variances, even for different ventilation systems. For P-UDAF ISO 5 class, there is a significant difference between the average of the measurements in Position 1 (operating table) and other positions. The variance of the measurements in Position 4 (anesthesia tower) is very high and it does not justify a significant difference in the average as compared to the other positions.

The surface bio-contamination values are very similar for both different ventilation types and for ISO classifications (ISO 5 and ISO 7) with a significant difference near the surgical table. At sampling points that are outside the surgical table, the values do not differ much between the ventilation systems. When comparing same ISO class OTs, e.g., ISO 7, the surface bio-contamination in Type C and Type D OTs is, on average, similar or lower than in OTs with Type A and Type B systems. Notwithstanding, surface contamination found in OTs with P-UDAF systems (Type A) ISO 5 class, with the exception of anesthesia tower (position 4), was well below the threshold values. In contrast, P-UDAF (Type B) ISO 7 class systems in this study always have values comparable to MAF (Type C and Type D) systems.

## 4. Limitations

The study has been conducted by monitoring some important environmental parameters in OTs by the same teams throughout a long inspection period. Although the measurements rely on INAIL, EU-GMP, and ISO methodologies, the differences encountered in the evaluated hospitals may set some limitations of this experimental study.

In “at-rest” conditions, the main limitation is the uncertainty about the cleaning procedure [26] and the unknown type and quality of the surface materials which may affect the final results [50]. In “operational” condition, limitations may arise due to the different influence of medical teams and patients. Humans, even if well gowned, may have different contamination emission rates. The fabric quality of the medical team gowning systems and the covering of equipment and surgical tools (e.g., cotton vs. polyester/TNT) can deeply influence the contamination emission rate [33], and affect the final contaminant concentrations. A further limitation may result from the different type of surgical operations monitored and the use of electrosurgical tools during operations [34,35,43].

## 5. Conclusions

The study presents the measurement results (both in “at-rest” conditions and “in operation) obtained in a large sample of Italian OTs and from inspections made during their operative life. The results confirm that, in general, the ventilation systems are able to obtain and maintain in their lifetime the intended cleanliness performance levels (classes). They attest that significant differences in real OT contamination control capabilities do exist and they could be ascribed to many design choices and different operation and maintenance practices. The study put forward and confirms that the type of air diffusion scheme and the design airflow rate are crucial determinants. Besides large variations in measurements, the average performance values, in terms of airborne particle and microbial contamination (in air and on surfaces), for P-UDAF systems (Type A and B), are better than the ones that represent the MAF air diffusion scheme that was evaluated in this campaign. Moreover, the average performances do increase with increasing airflows as shown by the Type A data vs. Type B, Type C, and Type D. The study suggests that the P-UDAF scheme is performing better, even when used with airflows (Type B), which are quite similar to the MAF (Type C and type D) ones. The MAF results are also showing that Type C (ceiling air diffusers) and Type D (high-wall diffusers) have quite similar performances; Type C solution have a slight advantage over Type D. The adopted design airflows (e.g., ACH) for all of the ventilation systems have relevant effects on energy consumptions, system complexity, and economic costs, but the analysis of obtained data shows that, in Type A systems, above a certain ACH level, the benefits of a further increase in airflow tend to be insignificant in terms of contamination reduction.

Microbial surface contamination in ventilation systems in “at-rest” conditions show that there is no inherent and univocal solution to limit contamination in OT fields, even though P-UDAF class ISO 5 OT have lower contamination value in the most protected (critical) area where it matters. The personnel crowd index and RT are useful performance indicators for evaluating the efficiency of a ventilation system to keep airborne contamination below a fixed limit in function of time, ventilation system, and personnel. From the point of view of the airborne particle contamination limit values, OTs with P-UDAF (Type A-ISO 5 and Type B-ISO 7) ventilation systems were compliant with the limit values imposed by Italian INAIL GL and Annex 1 EU-GMP 100% of the time; MAF systems, Type C and Type D, as well, reported 100% compliance rate, even if with less demanding limits.

OTs in Class ISO 5 that were equipped with P-UDAF systems had a non-compliance rate equal to 1.3% for the airborne microbial contamination limits in “operational conditions” according to INAIL GL. By applying the strict airborne microbial limits that were adopted by the Annex 1 of EU-GMP, P-UDAF systems had a non-compliance rate sensibly lower than MAF systems, 10.8% and 1.8% for ISO 5 Class (Grade B) and ISO 7 Class (Grade C), respectively, while MAF systems, which are ranked in Grade C, achieved a non-compliance rate equal to 20.3% and 10.1% for Class ISO 7 Type C and Type D, respectively.

In the scenario of a future increased presence of antibiotic resistant bacteria in OT, with a potential increase of SSIs, medical staff, HVAC designers, and healthcare management should pay more attention to the selection of the type of ventilation system and of the performance optimization in terms of contamination control and of energy and economic costs. In this contest, OTs equipped with P-UDAF system in ISO 5 class seem to be a more effective and stable solution for guarantying a low airborne bio-burden contamination during surgical activities for both patients and medical team. However, future investigation should further account for all of the variables, also the ones not explicitly accounted in this study.

## Figures and Tables

**Table 1:** Type of measurements and sampling locations according to the occupational state.

Qualification Test	Occupancy State	Positions
Microbiological air sampling	At-rest	Center of surgical table
Surface microbiological sampling	At-rest	4 surfaces in OT ^a^
Airborne particles sampling	At-rest	Protected zone for P-UDAF and everywhere for MAF systems ^b^
Recovery time	At-rest	Center of surgical table for MAF, dirtiest point outside ceiling filter for P-UDAF systems
Microbiological air sampling	Operational	Close to the surgical wound

^a^ surgical table, scialytic lamp, surgical instruments table, anesthesia tower. ^b^ sampling points under ceiling HEPA filter for ISO Class 5 OTs (P-UDAF) according to ISO 14644-1:2015 [46]; sampling points evenly distributed according to ISO 14644-1:2015 [46] for ISO Class 7 OTs (P-UDAF and MAF).

**Table 2:** Bio-contaminants threshold value for Operating Theatres (OTs) according INAIL GL [49].

**Microbiological Air Contamination Limits**			
At-Rest		Operational	
MAF	UDAF	MAF	UDAF
≤35 CFU m^−3^	NA	≤180 CFU m^−3^	≤20 CFU m^−3^
**Microbiological Surface Contamination Limits**			
≤5 CFU plate^−1^	≤5 CFU plate^−1^	NA	NA

**Table 3:** Particle and microbial threshold limit for clean area during operation according to Annex 1, EU-GMP [52].

EU-GMP Grade	ISO Class	Particle Concentration Limit (≥0.5 µm)		Microbial Concentration Limit	
		In Air, At-Rest [pp m^−3^]	In Air, Operational [pp m^−3^]	In Air, Operational [CFU m^−3^]	On Surface, Operational [CFU Plate^−1^]
A	5	3520	3520	<1	<1
B	5	3520	352,000	10	5
C	7	352,000	3,520,000	100	25
D	8	3,520,000	Not Defined	200	50

**Table 4:** Overview of the OTs and descriptive statistics of the measurement results.

System		P-UDAF		MAF	
OT Type		Type A	Type B	Type C	Type D
ISO Class		5	7	7	7
Number of OTs		27	31	16	101
Ambient Volume [m^3^]	Mean	136.9	126.0	120.0	105.4
	Standard deviation	13.3	16.0	22.6	22.5
ACH	Mean	51.2	19.0	15.1	18.4
	Standard deviation	5.5	3.8	5.0	5.2
Airborne particle concentration [pp m^−3^], (≥0.5 µm) in at-rest conditions	Mean	326	25,970	28,878	56,400
	Standard deviation	235	31,525	14,479	85,332
	Min	0	0	2027	437
	Max	2641	193,313	229,613	556,904
Microbiological air contamination [CFU m^−3^] in at-rest conditions	Mean	0.9	3.3	9.9	11.8
	Standard deviation	1.0	3.5	4.4	12.7
	Min	0.0	0.0	0.0	0.0
	Max	13.3	30.5	86.7	392.5
Microbiological air contamination [CFU m^−3^] in operational conditions	Mean	5.5	20.7	72.8	55.2
	Standard deviation	7.0	20.2	18.7	32.0
	Min	0.0	0.0	13.6	4.2
	Max	125.0	167.9	327.5	339.4
Recovery time [min]	Mean	7.2	14.0	28.5	23.0
	Standard deviation	0.6	4.1	11.9	8.4
	Min	6.2	8.5	18.3	11.0
	Max	7.7	20.8	66.7	40.8
Inspections non-compliant with INAIL [49] particle concentrations threshold values at-rest [%]		0	0	0	0
Inspections non-compliant with INAIL [49] airborne CFU threshold values at-rest [%]		NA	NA	2.6	6.1
Inspections non-compliant with INAIL [49] airborne CFU threshold values in operation [%]		1.3	23.2	3.1	2.4
Inspections non-compliant with EU-GMP [52] airborne CFU threshold values in operation [%]		10.8	1.8	20.3	10.1
		(Grade B)	(Grade C)	(Grade C)	(Grade C)

**Table 5:** Results of comparison between multiple OTs samples. The comparisons are referred to the 175 investigated OTs in terms of particle concentration “at-rest” and microbiological air contamination “at-rest” and “operational”.

Comparison		*p*-Value		
**Sample 1**	Sample 2	Particle Concentration “At-Rest” (Figure 1)	Microbiological Air Contamination “At Rest” (Figure 2)	Microbiological Air Contamination “Operational” (Figure 4)
Type A	Type B	0.568	0.795	0.133
Type A	Type C	0.522	0.016	0.000
Type A	Type D	0.001	0.000	0.000
Type B	Type C	0.997	0.105	0.000
Type B	Type D	0.225	0.000	0.000
Type C	Type D	0.513	0.968	0.030
